# Association Between Platelet Count/Lactate Dehydrogenase Ratio and In-Hospital Mortality in the Patients With Sepsis-Induced Myocardial Injury

**DOI:** 10.31083/RCM55645

**Published:** 2026-10-22

**Authors:** Shuai Mao, Xuan Zhang, Yunzhang Zhao, Chao Lv, Huiyi Liu, Yanzhu Yao, Yuyan Wang, Yifan Wang, Shengshu Wang, Jie Zhang, Yong Xu, Tong Yin

**Affiliations:** ^1^Institute of Geriatrics, National Clinical Research Center for Geriatric Diseases, The Second Medical Center of Chinese PLA General Hospital, 100853 Beijing, China; ^2^Medical School of Chinese PLA, Chinese PLA General Hospital, 100853 Beijing, China; ^3^Senior Department of Cardiology, The Sixth Medical Center of PLA General Hospital, 100048 Beijing, China; ^4^Department of Medical Innovation Research, Chinese PLA General Hospital, 100853 Beijing, China

**Keywords:** sepsis-induced myocardial injury, PLT/LDH ratio, in-hospital mortality, predictive value

## Abstract

**Background:**

Sepsis-induced myocardial injury (SIMI) is a severe complication with high mortality, yet effective prognostic prediction tools remain limited. Although platelet-derived indices have been explored in SIMI, these metrics primarily reflect systemic inflammation rather than the extent of myocardial injury or metabolic status.

**Methods:**

We retrospectively analyzed 3071 critically ill individuals with SIMI admitted to the intensive care unit (ICU) of the Chinese People’s Liberation Army (PLA) General Hospital between January 2020 and October 2025. The association between the platelet count (PLT)/lactate dehydrogenase (LDH) ratio and in-hospital mortality was assessed using multivariable Cox regression, restricted cubic splines, and receiver operating characteristic (ROC) curve analysis. Subgroup analyses evaluated consistency across clinically relevant strata.

**Results:**

The PLT/LDH ratio was independently associated with lower in-hospital mortality (hazard ratio [HR] = 0.44, 95% confidence interval [CI]: 0.34–0.57, *p* < 0.001). Compared with the lowest tertile, the highest tertile of PLT/LDH ratio was associated with a 67% reduction in risk (HR = 0.33, 95% CI: 0.25–0.43, *p* < 0.001). The PLT/LDH ratio demonstrated modest, statistically significant incremental discriminative performance over PLT or LDH alone (both *p* < 0.05). A nonlinear relationship was observed, with an inflection point at approximately 0.306 (*p* for nonlinearity < 0.0001). Subgroup analyses revealed a consistent association across all subgroups, with significant interactions for sex, hypertension, chronic kidney disease, and liver disease (all *p* for interaction < 0.05).

**Conclusions:**

The PLT/LDH ratio is independently associated with in-hospital mortality in patients with SIMI. It may serve as a simple and practical tool for prognostic prediction in patients with SIMI upon ICU admission.

## 1. Introduction

Sepsis remains a major contributor to mortality and morbidity in intensive care units (ICUs) worldwide, with septic shock representing its most severe manifestation [1]. As a common and serious complication, sepsis-induced myocardial injury (SIMI) is characterized primarily by transient myocardial depression, which can significantly worsen patient outcomes [2]. In patients with severe SIMI, myocardial dysfunction has been linked to mortality rates exceeding 80%, especially in those with septic shock [3]. Therefore, as a reversible pathophysiological process, the assessment of myocardial injury severity is crucial for determining the prognosis of patients with SIMI.

Recent studies have explored the value of platelet-derived indices in assessing SIMI [4]. The platelet count (PLT) to lymphocyte ratio (PLR) and PLT to neutrophil ratio (PNR) have been closely linked to myocardial injury in patients with sepsis [5]. However, these measures primarily reflect the host response to infection rather than the underlying metabolic state of the heart. Therefore, no platelet-based marker currently integrates information on both myocardial injury and systemic energy metabolism.

Lactate dehydrogenase (LDH) is an enzyme present in many cell types, and elevated blood levels reflect widespread cellular injury [6]. It has long been used as a marker of tissue damage and metabolic stress [7]. In the setting of SIMI, combining platelet count with LDH to form the PLT/LDH ratio represents a composite marker that integrates information on both platelet-related aspects and systemic metabolic status. The PLT/LDH ratio was specifically chosen because it integrates two biologically complementary dimensions. Platelet count reflects hemostatic competence, endothelial integrity, and innate immune function, whereas LDH serves as a sensitive indicator of tissue hypoxia, cellular necrosis, and global metabolic burden. Unlike platelet-based inflammatory markers such as PLR or PNR, which primarily reflect the host inflammatory response, the PLT/LDH ratio captures the balance between the host’s reparative capacity (platelets) and the extent of ongoing tissue destruction (LDH). This dual nature makes it conceptually more relevant to the pathophysiology of SIMI, a condition characterized by concurrent myocardial injury and systemic metabolic derangement. In this research, we evaluated the predictive value of the PLT/LDH ratio for in-hospital mortality in patients with SIMI, and also probed into its feasibility as an effective means for early risk stratification.

## 2. Materials and Methods

### 2.1 Study Population

This retrospective study enrolled patients with SIMI admitted to the ICU of Chinese People’s Liberation Army (PLA) General Hospital from January 2020 to October 2025. Inclusion criteria were that they met all of the following criteria: age ≥18 years, a confirmed diagnosis of sepsis according to the Sepsis-3 definitions [1], fulfillment of the SIMI criteria within 24 hours of ICU admission, and an ICU length of stay >24 hours and ≤100 days. To mitigate immortal-time bias, a 24-hour landmark approach was adopted, restricting inclusion to patients who survived the initial 24 hours post-ICU admission. Survival analysis was then initiated from this critical time point, ensuring the study population better reflects the broader SIMI cohort rather than those with early mortality. Patients who die within the first 24 hours often follow distinct clinical trajectories and may not be representative of the broader SIMI population for prognostic assessment, further justifying this design. The patient screening and inclusion process is summarized in **Supplementary Fig. 1**.

On the day of ICU admission, cTnT levels were measured, and the maximum value obtained that day was used to define SIMI. The upper reference limit (99th percentile) for cTnT at our institution is 0.01 ng/mL, and SIMI was defined as a cTnT level exceeding this threshold within 24 hours of admission [8,9,10]. We recognize that this definition identifies myocardial injury occurring during sepsis, but does not by itself confirm a diagnosis of sepsis-induced cardiomyopathy, which requires echocardiographic and hemodynamic assessment. Accordingly, we use the abbreviation SIMI throughout this manuscript to refer to this entity. To ensure the specificity of cardiac troponin T (cTnT) elevation, we systematically excluded participants with conditions known to precipitate cTnT release through direct myocardial injury or indirect hemodynamic stress. These exclusion criteria encompassed acute coronary syndrome, various forms of cardiomyopathy, inflammatory heart diseases (including myocarditis, endocarditis, and pericarditis), significant valvular pathology, chronic obstructive pulmonary disease, and chronic heart failure. Furthermore, individuals were disqualified if they had undergone cardiac surgery preceding ICU admission, experienced cardiac arrest, or possessed a history of severe tachyarrhythmias, specifically supraventricular tachycardia, ventricular tachycardia, ventricular fibrillation, or ventricular flutter. The study population was further refined by excluding pregnant women and patients diagnosed with congenital coagulopathies, such as hemophilia, hereditary fibrinogen deficiency, or other inherited coagulation factor deficits.

### 2.2 Data Collection

For each patient, comprehensive clinical data from the first 24 hours of ICU admission were extracted, including demographics, comorbidities, and laboratory parameters. The laboratory panel encompassed hematological indices (white blood cell count [WBC], red blood cell count [RBC], PLT, hemoglobin [HGB], fibrinogen, D-dimer), coagulation parameters (prothrombin time [PT], international normalized ratio [INR]), inflammatory markers (interleukin-6 [IL-6], C-reactive protein [CRP]), liver function tests (alanine aminotransferase [ALT], aspartate aminotransferase [AST]), myocardial injury markers (cTnT, myoglobin, creatine kinase-MB [CK-MB], LDH), renal function indicators (blood urea nitrogen [BUN], serum creatinine [Scr]), and lactate. The PLT/LDH ratio was calculated as PLT (×10^9^/L) divided by LDH (U/L), using the first available values within 24 hours of admission, which in most cases were obtained before the initiation of vasopressor therapy or blood product transfusions.

### 2.3 Outcomes of This Study

The principal endpoint assessed was mortality occurring during hospitalization, whereas the secondary endpoint focused on death within 28 days. Survival time for both outcomes was calculated from the 24-hour landmark, and patients alive at the end of follow-up were censored at the corresponding time point (discharge for in-hospital mortality, or 28 days for 28-day mortality).

### 2.4 Statistical Analysis

Continuous variables were presented as mean ± standard deviation (SD) or median with interquartile range (IQR), depending on the normality of distribution, and were compared using Student’s *t*-test or the Mann-Whitney U test as appropriate. Categorical variables were presented as counts (proportions) and compared using the chi-square test or Fisher’s exact test as needed.

To assess the relationship between the PLT/LDH ratio and in-hospital mortality, we employed Multifactorial Cox proportional hazards regression models, with the outcomes presented as hazard ratios (HR) and 95% confidence intervals (CI). These models were adjusted for potential confounders including age, gender, Sequential Organ Failure Assessment (SOFA) score, cardiac troponin T, myoglobin, and creatine kinase-MB. To further investigate the nature of the dose-response association, restricted cubic splines (RCS) with three knots were integrated into the Cox model. To evaluate the predictive ability of the PLT/LDH ratio, receiver operating characteristic (ROC) curves were employed, with the area under the curve (AUC) computed; DeLong’s test was then used to compare these AUC values. Subgroup analyses were performed according to prespecified clinically relevant variables, including age (<65 vs. ≥65 years), sex, history of coronary artery disease (CAD), hypertension, chronic kidney disease (CKD), diabetes mellitus (DM), liver disease, SOFA score (<10 vs. ≥10), and Charlson comorbidity index (<5 vs. ≥5). Interaction effects were assessed by introducing product terms between the PLT/LDH ratio and each stratification variable into the Multifactorial Cox regression models. Missing data were handled by complete‑case analysis, as the proportion of missing values was <5% for each included variable. Sensitivity analyses using multiple imputation (chained equations, 5 imputations) yielded consistent results in terms of effect estimates and statistical significance (data not shown). All statistical tests were two-sided, and a *p* value < 0.05 was considered statistically significant. Statistical analyses were performed using R software (version 4.2.3; R Foundation for Statistical Computing, Vienna, Austria).

## 3. Results

### 3.1 Baseline Characteristics of Patients With SIMI

A total of 3071 patients with SIMI were recruited from the ICU electronic medical record database of the Chinese People’s Liberation Army (PLA) General Hospital. Table 1 summarizes the baseline demographic and clinical profiles for both cohorts. Based on in-hospital outcomes, patients were divided into an in-hospital survivor group (n = 2508) and an in-hospital non-survivor group (n = 563), with an in-hospital mortality rate of 20.51%.

**Table 1.** **Baseline characteristics of SIMI**.

Characteristics		Hospital survivors (n = 2710)	Hospital non-survivors (n = 680)	*p* value
Age, mean (SD)		60.489 (19.087)	72.582 (17.087)	<0.001
Gender				0.679
	Female, n (%)	894 (32.989)	230 (33.824)	
	Male, n (%)	1816 (67.011)	450 (66.176)	
Comorbidity				
	Hypertension, n (%)	959 (35.387)	166 (24.412)	<0.001
	DM, n (%)	551 (20.332)	111 (16.324)	0.018
	CAD, n (%)	351 (12.952)	111 (16.324)	0.022
Laboratory examination				
	WBC count (10^9^/L), median (IQR)	9.350 (8.100–9.850)	9.530 (8.585–9.940)	<0.001
	RBC count (10^12^/L), mean (SD)	3.708 (0.743)	3.638 (0.848)	0.034
	PLT (10^9^/L), median (IQR)	98.000 (90.000–265.750)	93.000 (72.250–99.000)	<0.001
	PT (s), median (IQR)	16.900 (14.900–21.400)	22.700 (18.400–34.700)	<0.001
	INR, median (IQR)	1.370 (1.180–1.850)	1.970 (1.540–3.265)	<0.001
	CRP (mg/dL), median (IQR)	7.194 (2.241–9.316)	8.825 (5.970–9.768)	<0.001
	IL-6 (ng/L), median (IQR)	3.993 (0.991–31.370)	0.661 (0.020–1.622)	<0.001
	ALT (U/L), median (IQR)	21.000 (9.300–78.750)	27.300 (9.200–87.925)	<0.001
	AST (U/L), median (IQR)	46.450 (16.475–82.600)	73.350 (36.925–95.775)	<0.001
	BUN (mg/dL), median (IQR)	9.570 (8.318–16.250)	9.650 (8.655–26.290)	<0.001
	SCR (μmol/L), median (IQR)	96.800 (79.800–365.600)	95.900 (84.200–204.925)	<0.001
	Lac (mmol/L), median (IQR)	2.800 (1.900–4.770)	7.300 (4.400–9.300)	<0.001
	LDH (U/L), median (IQR)	359.350 (255.550–587.275)	588.600 (360.250–860.850)	<0.001
	HGB (g/L), median (IQR)	98.000 (94.000–99.000)	98.000 (93.000–99.000)	0.015
	Fibrinogen (g/L), median (IQR)	5.010 (3.835–6.245)	4.800 (3.215–6.190)	<0.001
	D-dimer (mg/L), median (IQR)	6.230 (2.748–9.370)	8.550 (4.840–20.000)	<0.001
	cTnT (ng/mL), median (IQR)	0.077 (0.034–0.183)	0.184 (0.088–0.426)	<0.001
	Myoglobin (ng/mL), median (IQR)	87.900 (31.700–169.700)	233.000 (83.000–883.900)	<0.001
	CK-MB (μg/L), median (IQR)	3.080 (1.390–6.268)	4.830 (2.465–8.285)	<0.001
Critical assessment on admission				
	SOFA score, median (IQR)	4.000 (4.000–6.000)	6.000 (4.000–8.000)	<0.001
	Charlson Comorbidity Index, median (IQR)	2.000 (0.000–3.000)	3.000 (0.000–3.000)	<0.001

Data are presented as mean ± standard deviation (SD) or median (interquartile range, IQR) for continuous variables, and as number (%) for categorical variables. Group comparisons were performed using Student’s *t*-test or Mann-Whitney U test for continuous variables, and the chi-square test or Fisher’s exact test for categorical variables, as appropriate. SIMI, sepsis-induced myocardial injury; cTnT, cardiac troponin T; WBC, white blood cell count; RBC, red blood cell count; PLT, platelet count; PT, prothrombin time; INR, international normalized ratio; CRP, C-reactive protein; IL-6, interleukin-6; ALT, alanine aminotransferase; AST, aspartate aminotransferase; BUN, blood urea nitrogen; SCR, serum creatinine; Lac, lactate; LDH, lactate dehydrogenase; HGB, hemoglobin; CKMB, creatine kinase-MB; SOFA, Sequential Organ Failure Assessment; DM, diabetes mellitus; CAD, coronary artery disease.

Relative to survivors, non-survivors exhibited a markedly advanced age (72.58 ± 17.09 vs. 60.49 ± 19.09 years, *p* < 0.001). Regarding comorbidities, the in-hospital non-survivor group had lower rates of hypertension and diabetes (both *p* < 0.05), but a higher rate of coronary artery disease (16.32% vs. 12.95%, *p* = 0.022). Laboratory findings showed that non-survivors had significantly higher levels of inflammatory markers (CRP) and myocardial injury markers (cTnT, myoglobin, CK-MB, and LDH), as well as higher levels of liver injury markers (ALT, AST), coagulation markers (INR, D-dimer), and lactate (all *p* < 0.001). However, IL-6 levels were significantly lower in non-survivors than in survivors (median 0.66 vs. 3.99 ng/L, *p* < 0.001), a finding that may reflect the immunoparalysis phase frequently observed in the later stages of sepsis, while **Supplementary Table 1** stratifies patients by PLT/LDH ratio tertiles. These two grouping strategies capture distinct disease dimensions, and the divergent IL‑6 trends are therefore not contradictory. Platelet count was also significantly lower in non-survivors (median 93.00 vs. 98.00 × 10^9^/L, *p* < 0.001). In addition, disease severity scores (SOFA score and Charlson Comorbidity Index) were significantly higher in non-survivors (both *p* < 0.001).

### 3.2 Association Between PLT/LDH Ratio and In-Hospital Mortality in Patients With SIMI

In the unifactorial Cox regression, a higher PLT/LDH ratio was significantly associated with a lower risk of in-hospital mortality (HR = 0.28, 95% CI: 0.21–0.36, *p* < 0.001). After multifactorial adjustment for age, sex, SOFA score, cardiac troponin T, myoglobin, and creatine kinase-MB, the PLT/LDH ratio remained independently associated with lower mortality (HR = 0.44, 95% CI: 0.34–0.57, *p* < 0.001) (Table 2). Based on the PLT/LDH ratio, all patients were divided into tertiles: low (≤0.19), intermediate (0.19–0.59), and high (≥0.59). Compared to the low ratio group, patients in the high ratio group exhibited significantly lower levels of inflammatory markers (CRP, IL-6), less severe myocardial injury (cTnT, myoglobin, CK-MB), and SOFA score (all *p* < 0.001) (**Supplementary Table 1**).

**Table 2.** **Association between PLT/LDH ratio and in-hospital mortality in patients with SIMI**.

Variables		Univariable			Multifactorial		
		HR	95% CI	*p* value	HR	95% CI	*p* value
PLT/LDH		0.28	0.21–0.36	<0.001	0.44	0.34–0.57	<0.001
PLT/LDH tertile							
	Low	Ref			Ref		
	Middle	0.44	0.36–0.54	<0.001	0.46	0.38–0.57	<0.001
	High	0.24	0.19–0.30	<0.001	0.33	0.25–0.43	<0.001

Hazard ratios (HRs) and 95% CIs were estimated using Cox proportional hazards regression models. Multifactorial models were adjusted for age, gender, SOFA score, cardiac troponin T, myoglobin, and creatine kinase-MB.

Compared with the low tertile group, the middle tertile group had a 54% reduction in mortality risk (HR = 0.46, 95% CI: 0.38–0.57, *p* < 0.001), and the high tertile group showed a 67% reduction (HR = 0.33, 95% CI: 0.25–0.43, *p* < 0.001) in the fully adjusted model (Table 2). These results indicate a stepwise negative correlation between the PLT/LDH ratio and in-hospital mortality among patients with SIMI.

Kaplan-Meier curves (Fig. 1) revealed significant differences in in-hospital survival rates among the three groups (*p* < 0.001). Patients in the high PLT/LDH ratio group had significantly higher survival rates compared to those in the middle and low ratio groups, indicating a strong association between a higher PLT/LDH ratio and improved in-hospital survival in patients with SIMI.

**Fig. 1.**
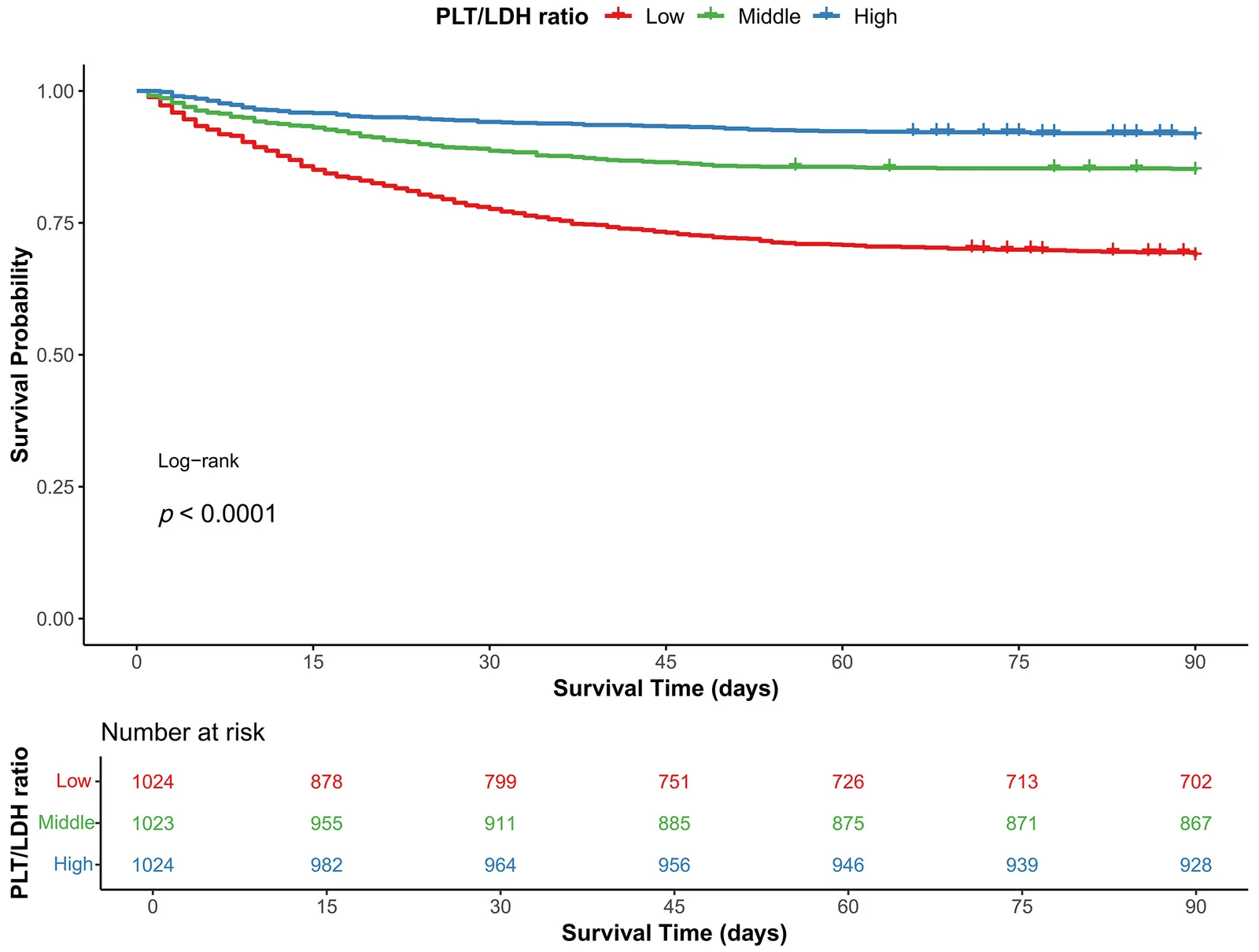
**Kaplan-Meier survival curves for in-hospital mortality in patients with SIMI**. The curves correspond to the three groups stratified by PLT/LDH ratio: low (red line), middle (green line), and high (blue line). PLT, platelet count.

Given the possibility of informative censoring caused by hospital discharge, we additionally evaluated 28-day mortality as a predefined endpoint. The PLT/LDH ratio remained significantly associated with lower 28-day mortality (HR = 0.387, 95% CI: 0.277–0.539, *p* < 0.001). Compared with the lowest tertile, the middle tertile (HR = 0.500, 95% CI: 0.391–0.640, *p* < 0.001) and highest tertile (HR = 0.336, 95% CI: 0.244–0.462, *p* < 0.001) showed progressively lower risks. These results align with the main analysis focusing on in-hospital mortality.

### 3.3 Predictive Performance of PLT/LDH Ratio for In-Hospital Mortality

Analysis of ROC curves revealed that the AUC for the PLT/LDH ratio in predicting in-hospital mortality was 0.696 (95% CI: 0.672–0.72). In addition, the PLT/LDH ratio demonstrated a comparable yet statistically meaningful enhancement in its ability to distinguish between patients with in-hospital mortality risk when compared to using PLT or LDH individually (Fig. 2, **Supplementary Table 2**).

**Fig. 2.**
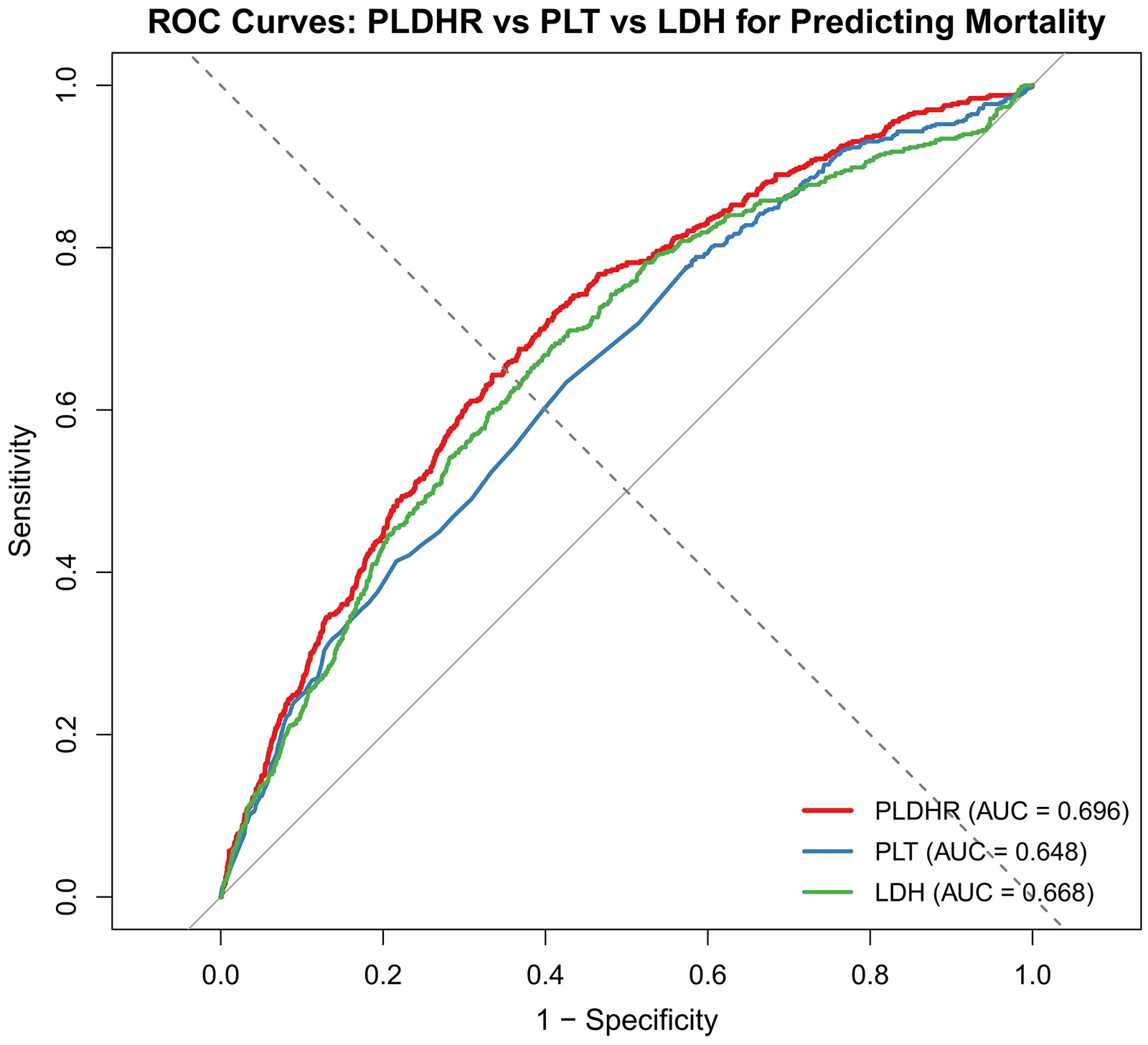
**Receiver operating characteristic (ROC) curves for the prediction of in-hospital mortality by PLT/LDH ratio (red line), PLT (blue line), and LDH (green line) in patients with SIMI**. The area under the curve (AUC) with 95% confidence intervals was calculated to assess discriminative ability.

Incremental prognostic value beyond the SOFA-based model: To assess whether the PLT/LDH ratio provides additional prognostic information beyond an established clinical model, we developed a baseline model incorporating age, sex, and SOFA score. The addition of PLT/LDH significantly improved the model’s discriminative performance (C-index: from 0.726 [95% CI: 0.700–0.752] to 0.744 [95% CI: 0.718–0.770]; *p* < 0.001 by likelihood ratio test). The continuous net reclassification improvement (NRI) was 0.0267. These findings are detailed in the **Supplementary Table 3**.

To examine the relationship pattern between the PLT/LDH ratio and mortality risk, a Cox proportional hazards model with RCS was applied, adjusting for age, gender, SOFA score, and Charlson Comorbidity Index. A significant nonlinear relationship was observed (*p* for nonlinearity < 0.0001). The hazard ratio for mortality decreased sharply with increasing PLT/LDH ratio up to a value of approximately 0.306, beyond which the curve plateaued, suggesting that further increases in the ratio were not associated with additional risk reduction. We emphasize that this value represents the point at which the HR crossed 1 relative to the chosen reference and should not be interpreted as a definitive biological cutoff or clinical threshold (Fig. 3, **Supplementary Table 4**).

**Fig. 3.**
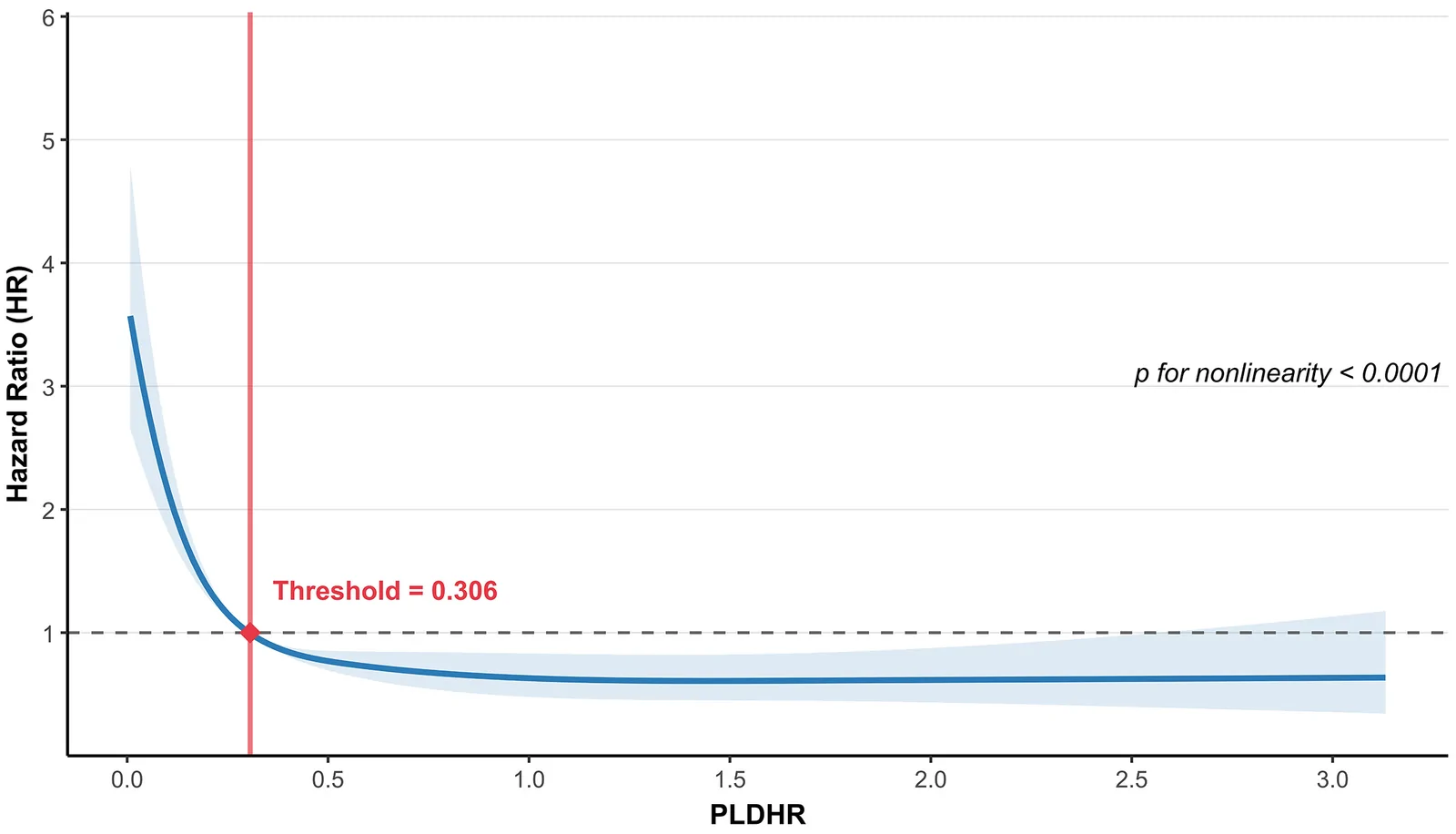
**Restricted cubic spline (RCS) analysis of the association between PLT/LDH ratio and in-hospital mortality risk**. Cox proportional hazards model was adjusted for age, gender, SOFA score, and Charlson comorbidity index. The red vertical line indicates the threshold value of the PLT/LDH ratio (0.306), where the HR equals 1 (dashed horizontal line). The shaded area represents the 95% confidence interval. A significant nonlinear relationship was observed (*p* for nonlinearity < 0.0001), with HR decreasing sharply as PLT/LDH ratio increases below the threshold and stabilizing near 1 above the threshold.

### 3.4 Subgroup Analysis

To further evaluate the association between PLT/LDH ratio and in-hospital mortality, subgroup analyses were performed across strata defined by age, sex, history of CAD, hypertension, CKD, DM, liver disease, SOFA score, and Charlson comorbidity index (Fig. 4). Overall, a consistent inverse association between PLT/LDH ratio and mortality was observed across most subgroups. The interaction terms for age, CAD, DM, SOFA score, and Charlson comorbidity index were not statistically significant (all *p* > 0.05), indicating that the association between PLT/LDH ratio and mortality remained stable across these subgroups. In contrast, significant interactions were observed for sex (*p* = 0.01), hypertension (*p* < 0.0001), CKD (*p* = 0.02), and liver disease (*p* = 0.04), suggesting that the protective effect of PLT/LDH ratio varied across these strata. Specifically, the protective association appeared more pronounced in females, patients with hypertension, and those with a history of CKD or liver disease. Collectively, these subgroup analyses indicate that the inverse association between PLT/LDH ratio and in-hospital mortality is generally consistent across most patient subsets, with some heterogeneity observed in specific subgroups.

**Fig. 4.**
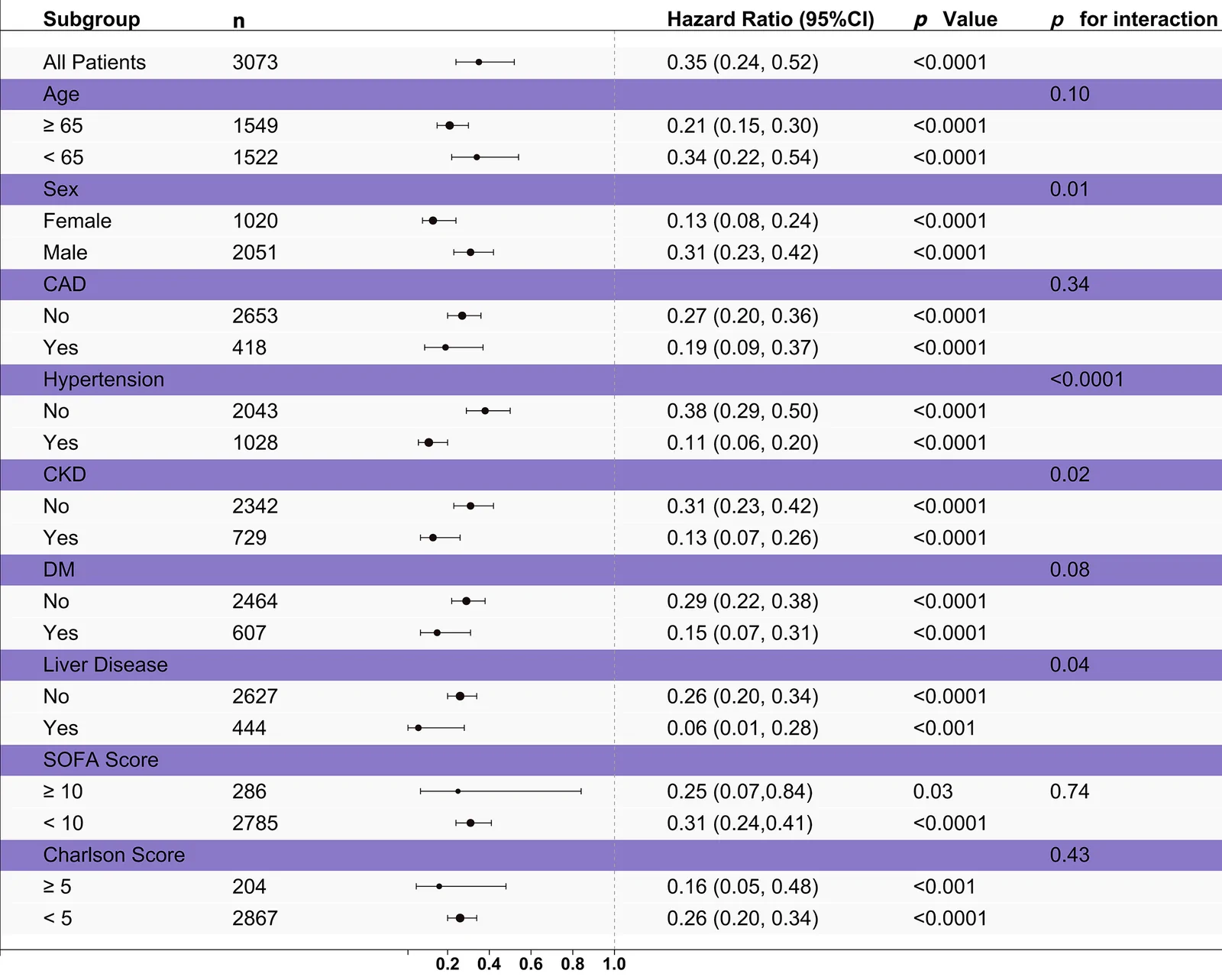
**Subgroup analysis of the relationship between PLT/LDH ratio and in-hospital mortality in SIMI patients**. Multifactorial Cox proportional hazards models, adjusted for age, gender, coronary artery disease, hypertension, chronic kidney disease, diabetes mellitus, SOFA score and Charlson comorbidity index score, were fitted within each subgroup. CKD, chronic kidney disease.

To further assess the robustness of our findings, we performed several additional stratification analyses. Stratification by cTnT elevation magnitude (low: ≤5 × URL, n = 1018; moderate: 5–20 × URL, n = 1238; high: >20 × URL, n = 815) showed consistent inverse associations in the moderate (HR = 0.343, 95% CI: 0.223–0.527, *p* <0.001) and high (HR = 0.338, 95% CI: 0.198–0.574, *p* <0.001) groups, whereas no significant association was observed in the low group (HR = 0.923, 95% CI: 0.668–1.277, *p* = 0.629), with a significant interaction across strata (*p* = 0.008). Stratification by serum creatinine tertiles (T1: ≤80.7 μmol/L, n = 753; T2: 80.7–96.6 μmol/L, n = 768; T3: >96.6 μmol/L, n = 1517) showed significant associations in T1 (HR = 0.673, 95% CI: 0.454–0.999, *p* = 0.049) and T3 (HR = 0.206, 95% CI: 0.124–0.343, *p* < 0.001), with a significant interaction across tertiles (*p* = 1.6 × 10^–4^). These results are presented in the **Supplementary Tables 5**,**6**.

## 4. Discussion

In this retrospective analysis, we found that the PLT/LDH ratio serves as an independent predictor of in-hospital mortality in patients with SIMI and a meaningful prognostic marker. Our results indicated that the PLT/LDH ratio exhibited a moderate yet statistically significant enhancement in predictive ability when compared to using PLT or LDH individually. These results indicate that the PLT/LDH ratio could act as a useful index for the initial risk classification and tailored care of SIMI patients, and it also functions as an independent factor linked to better survival outcomes in SIMI.

As a crucial enzyme in glycolysis, LDH is essential for cellular metabolism, primarily aiding the transformation of pyruvate into lactate. In clinical settings, increased LDH concentrations are associated with scenarios like ischemia, hypoxia, and inflammatory processes, typically serving as a measure of the degree of tissue harm. Therefore, LDH shows promise as a prognostic indicator for the early identification of serious illnesses including infections, tumours, and blood-related conditions, which frequently involve substantial tissue or cellular injury [11,12,13,14].

Platelets exhibit functional duality in sepsis: excessive activation promotes microthrombus formation and aggravates tissue ischemia [15], while they also act as immune sensors, releasing chemokines to recruit neutrophils and preserving endothelial barrier integrity [16]. Thrombocytopenia may thus reflect either consumptive coagulopathy or reduced microcirculatory embolic risk, illustrating this dual nature. Moreover, beyond the traditionally recognized mechanisms of consumptive reduction and bone marrow suppression, thrombocytopenia also signifies severe impairment of innate immune defense and vascular repair capacity [17,18], but these multidimensional pathological implications cannot be fully captured by a single platelet count.

Given the respective limitations of LDH and platelet count as single markers, integrating these two parameters into a composite indicator, the PLT/LDH ratio, may offer a more comprehensive assessment by simultaneously capturing tissue injury burden and platelet-related functional status. This notion is further supported by the fact that the concurrent occurrence of thrombocytopenia and elevated LDH is commonly seen in conditions such as hematologic malignancies [19,20], hemophagocytic lymphohistiocytosis (HLH) [21], and thrombotic microangiopathy (TMA) [22,23], where the coexistence of low platelets and high LDH reflects, from different perspectives, a simultaneous attack on the hematopoietic system and tissue integrity. In HLH, overactivated immune cells and the ensuing cytokine storm both consume platelets through phagocytosis and bone marrow suppression, while causing widespread tissue damage that leads to LDH release [24]. In TMA, widespread microvascular platelet thrombi not only consume large numbers of platelets, resulting in thrombocytopenia [25], but also cause tissue ischemia and red blood cell fragmentation due to vascular obstruction, leading to sharp increases in LDH [26]. In hematologic malignancies, tumor cell infiltration into the bone marrow directly suppresses megakaryocyte production and platelet generation [27], while the high metabolic activity, spontaneous apoptosis, or rapid lysis induced by treatment serve as major sources of LDH [19]. Therefore, the paradoxical changes in platelets and LDH essentially represent an external manifestation of the dynamic imbalance between the intensity of the disease process and the hematopoietic system reserve and consumption. The core principle is that regardless of whether inflammation, microthrombosis, or tumor infiltration dominates the pathological process, all simultaneously disrupt hematopoietic homeostasis and tissue integrity. In the context of SIMI, a similar pathophysiological synergy may occur, where myocardial injury and systemic metabolic disturbance converge. Although LDH is less specific than cardiac troponin as a marker of myocardial injury [28], its ability to reflect widespread tissue damage and metabolic derangement may give it greater comprehensive value in assessing overall disease severity and long-term prognosis in patients with sepsis-induced myocardial injury [29]. This also helps explain why the predictive performance of the PLT/LDH ratio exceeds that of other platelet or myocardial injury markers.

Mechanistically, a declining PLT/LDH ratio may reflect the synergistic worsening of two interconnected pathways: energy metabolic failure and impaired repair capacity. Initial myocardial injury leads to reduced cardiac output and tissue hypoperfusion, exacerbating systemic tissue hypoxia and mitochondrial dysfunction. This drives cells toward anaerobic metabolism with LDH release on one hand, while impairing platelet energy supply and accelerating their consumption and dysfunction on the other. The resulting thrombocytopenia further weakens microcirculatory flow regulation, worsening ischemia, hypoxia, and metabolic disturbances in the myocardium and other tissues. This vicious cycle ultimately propels patients from focal myocardial injury to widespread tissue energy failure and irreversible multiple organ dysfunction [30]. Thus, the PLT/LDH ratio may serve as a window into this underlying pathophysiological process, capturing the transition from compensated injury to systemic energy collapse. We emphasize that this mechanistic interpretation is speculative and hypothesis‑generating, requiring validation in experimental models.

Finally, to address potential concerns about the redundancy of the ratio, we performed sensitivity analyses in which PLT and LDH were included as individual restricted cubic spline terms. The addition of the PLT/LDH ratio as a linear term did not significantly improve model fit. This finding is not unexpected, as the ratio represents a linear combination of its two components. When the individual components are modeled flexibly with splines, the added contribution of a linear term is mathematically limited. Importantly, this does not diminish the clinical value of the PLT/LDH ratio as a simple and practical bedside marker.

### Limitations

Due to its retrospective nature and data collection approach, this research carries certain unavoidable limitations. First, as a single-center retrospective study, our results might lack broad applicability to other patient groups or medical environments, requiring further verification through larger, multi-center studies. Second, the clinical status of critically ill patients is dynamic and may fluctuate with disease progression. The present study, based on retrospective data, used only the PLT/LDH ratio measured at ICU admission and did not capture its temporal changes during the course of illness. Third, the absence of echocardiographic data precluded assessment of cardiac functional status and more precise stratification of myocardial injury severity; the diagnosis of SIMI relied solely on cTnT elevation, and future studies incorporating cardiac imaging will be essential to confirm these findings. Fourth, our adjustment strategy was limited by the availability of covariates; data on vasopressor use, mechanical ventilation, infection source, DIC indices, malignancy, and CRRT were not available for analysis.

## 5. Conclusions

Within this retrospective analysis, we established the PLT/LDH ratio as a standalone indicator of in-hospital fatality among individuals with SIMI, demonstrating improved predictive value over either PLT or LDH measured separately. This ratio could potentially act as an easy-to-use and effective method for assessing risk in this group of patients.

## Data Availability

The clinical data used in this study were obtained from the electronic medical record system of the Chinese PLA General Hospital. Due to institutional regulations and patient confidentiality, the data are not publicly available. Requests for access should be directed to the corresponding authors, subject to institutional approval.
