# Supplementary material for: Association Between Platelet Count/Lactate Dehydrogenase Ratio and In-Hospital Mortality in the Patients With Sepsis-Induced Myocardial Injury
Source: Rev Cardiovasc Med. 2026 Oct 22;27(10):55645. doi: 10.31083/RCM55645 (PMC13732677; doi:10.31083/RCM55645)
Supplement: Supplementary file 1 [file 2153-8174-27-10-55645-s1.zip › Supplementary Material.docx]

**Supplementary Table 1. Baseline characteristics of patients with SIMI stratified by PLT/LDH ratio tertiles.**

| **Characteristics** | **Low**  **(n = 1,024)** | **Middle**  **(n = 1,023)** | **High**  **(n = 1,024)** | ***p* value** |
| --- | --- | --- | --- | --- |
| **Age, mean (SD)** | 60.845 (18.575) | 66.272 (18.732) | 60.871 (20.060) | <0.001 |
| **Gender** |  |  |  | <0.001 |
| Female, n (%) | 385 (37.598) | 338 (33.040) | 297 (29.004) |  |
| Male, n (%) | 639 (62.402) | 685 (66.960) | 727 (70.996) |  |
| **Comorbidity** |  |  |  |  |
| Hypertension, n (%) | 200 (19.531) | 166 (24.412) | 443 (43.262) | <0.001 |
| DM, n (%) | 141 (13.770) | 217 (21.212) | 249 (24.316) | <0.001 |
| CAD, n (%) | 102 (9.961) | 150 (14.663) | 166 (16.211) | <0.001 |
| **Laboratory examination** |  |  |  |  |
| WBC count (10^9^/L), median (IQR) | 9.460  (8.362-9.900) | 9.530  (8.585-9.940) | 9.315  (8.050-9.830) | <0.001 |
| RBC count (10^12^/L), median (IQR) | 3.570  (3.120-4.140) | 3.590  (3.180-4.090) | 3.715  (3.200-4.220) | 0.025 |
| PLT (10^9^/L), median (IQR) | 87.000  (60.000-96.000) | 96.000  (90.000-112.000) | 289.000 (209.000-385.500) | <0.001 |
| PT (s), median (IQR) | 21.600  (17.600-32.600) | 17.000  (15.200-21.025) | 15.800  (14.400-18.000) | <0.001 |
| INR, median (IQR) | 1.870  (1.440-3.060) | 1.390  (1.200-1.810) | 1.260  (1.120-1.485) | <0.001 |
| CRP (mg/dL), median (IQR) | 8.512  (5.075-9.670) | 7.360  (2.851-9.349) | 6.192  (1.140-9.007) | <0.001 |
| IL-6 (ng/L), median (IQR) | 95.920  (59.240-1000.000) | 65.540  (8.398-95.562) | 20.700  (7.075-87.715) | <0.001 |
| ALT (U/L), median (IQR) | 57.500  (9.600-93.950) | 15.700  (9.200-66.400) | 9.900  (8.900-55.800) | <0.001 |
| AST (U/L), median (IQR) | 82.500  (51.075-97.200) | 42.300  (18.300-75.000) | 28.850  (9.800-62.625) | <0.001 |
| BUN (mg/dL), median (IQR) | 9.590  (8.612-9.970) | 9.560  (8.335-21.285) | 9.600  (8.210-19.885) | 0.146 |
| SCR (μmol/L), median (IQR) | 95.100  (82.750-159.475) | 97.100  (83.650-365.525) | 98.600  (74.875-442.125) | <0.001 |
| Lac (mmol/L), median (IQR) | 5.235  (2.900–8.275) | 2.700  (1.890–4.500) | 2.400  (1.700–3.425) | <0.001 |
| LDH (U/L), median (IQR) | 756.400  (568.450-926.500) | 346.800  (271.800-451.150) | 261.400  (189.000-337.150) | <0.001 |
| HGB (g/L), median (IQR) | 97.699 (18.077) | 101.689 (20.404) | 102.360 (22.105) | <0.001 |
| Fibrinogen (g/L), mean (SD) | 4.788 (1.915) | 4.911 (1.798) | 5.411 (1.639) | <0.001 |
| D-dimer (mg/L), median (IQR) | 9.215  (5.900-20.000) | 5.980  (2.900-8.950) | 4.155  (2.070-7.715) | <0.001 |
| cTnT (ng/mL), median (IQR) | 0.151  (0.061-0.401) | 0.087  (0.036-0.189) | 0.059  (0.027-0.131) | <0.001 |
| Myoglobin (ng/mL), median (IQR) | 97.650  (61.375-857.500) | 92.850  (57.150-265.700) | 79.200  (21.000-98.800) | <0.001 |
| CK-MB (μg/L), median (IQR) | 5.360  (2.185-8.668) | 3.235  (1.548-5.785) | 2.340  (1.220-4.540) | <0.001 |
| **Critical assessment on admission** |  |  |  |  |
| SOFA score, median (IQR) | 6.000  (5.000-8.000) | 5.000  (3.000-6.000) | 4.000  (3.000-4.000) | <0.001 |
| CHARLSON score, median (IQR) | 2.000  (0.000-3.000) | 2.000  (0.000-3.000) | 2.000  (0.000-3.000) | 0.006 |

Data are presented as mean ± standard deviation (SD) or median (interquartile range, IQR) for continuous variables, and as number (%) for categorical variables. Group comparisons were performed using Student's t-test or Mann-Whitney U test for continuous variables, and the chi-square test or Fisher's exact test for categorical variables, as appropriate. SIMI, sepsis-induced myocardial injury; cTnT, cardiac troponin T; WBC, white blood cell count; RBC, red blood cell count; PLT, platelet count; PT, prothrombin time; INR, international normalized ratio; CRP, C-reactive protein; IL-6, interleukin-6; ALT, alanine aminotransferase; AST, aspartate aminotransferase; BUN, blood urea nitrogen; Scr, serum creatinine; Lac, lactate; LDH, lactate dehydrogenase; HGB, hemoglobin; CK-MB, creatine kinase-MB; SOFA, Sequential Organ Failure Assessment.

**Supplementary Table 2. Area under the curve (AUC) of PLT/LDH ratio, PLT, and LDH for predicting in-hospital mortality**

| **Variables** | **AUC (95% CI)** | ***p* value** |
| --- | --- | --- |
| PLT/LDH | 0.696 (0.672-0.72) |  |
| PLT | 0.648 (0.623-0.672) | <0.001 |
| LDH | 0.668 (0.643-0.693) | 0.002 |

Comparisons of AUCs were performed using the DeLong test. *P* values indicate statistical significance of the difference in AUC between the PLT/LDH ratio and each individual marker. PLT: platelet count; LDH: lactate dehydrogenase.

**Supplementary Table 3. Model performance metrics comparing base model with and without PLT/LDH ratio**

| Model | C-index (95% CI) | Δ C-index | IDI (95% CI) | NRI | Brier Score (28-day) | Calibration Slope |
| --- | --- | --- | --- | --- | --- | --- |
| Base (Age + Sex + SOFA) | 0.726 (0.700-0.752) | — | — | — | 0.1370 | 1.000 |
| Base + PLT/LDH ratio | 0.744 (0.718-0.770) | +0.018 | 0.0093 (0.0046-0.0152) | 0.0267 | 0.1367 | 0.996 |
| *p* value | — | <0.001 | <0.001 | — | — | — |

Abbreviations: IDI, integrated discrimination improvement; NRI, net reclassification improvement; CI, confidence interval.
Notes: C-index values were calculated using Somers' D statistic with 1,000 bootstrap resamples for optimism correction. Brier scores and calibration slopes were evaluated at 28 days. Calibration slopes close to 1.0 indicate good calibration for both models.IDI and NRI were calculated at the 28-day time point using the following risk categories: low (<0.2), moderate (0.2-0.5), and high (>0.5).

**Supplementary Table 4. Comparison of the PLT/LDH ratio with PLT and LDH as separate nonlinear terms**

| **Model** | **Log-likelihood** | **C-index** | **LRT χ²** | **df** | ***p* value** |
| --- | --- | --- | --- | --- | --- |
| PLT + LDH (RCS, 3 knots each) + covariates | −4,221.8 | 0.621 | — | — | — |
| PLT + LDH (RCS) + PLT/LDH ratio + covariates | −4,221.6 | 0.621 | 0.31 | 1 | 0.578 |

Abbreviations: RCS, restricted cubic splines; LRT, likelihood ratio test; df, degrees of freedom.
Covariates: age, sex, SOFA score, Charlson comorbidity index.
Note: C-index values reflect the discriminative performance of the linear predictor from each model. The LRT compares the model with PLT/LDH ratio against the model without it.

**Supplementary Table 5. cTnT-stratified analysis of the association between PLT/LDH ratio and in-hospital mortality**

| **Stratum** | **n** | **HR (95% CI)** | ***p* value** |
| --- | --- | --- | --- |
| Low (≤5× URL) | 1,018 | 0.923 (0.668-1.277) | 0.629 |
| Moderate (5-20× URL) | 1,238 | 0.343 (0.223-0.527) | <0.001 |
| High (>20× URL) | 815 | 0.338 (0.198-0.574) | <0.001 |

Interaction *P* value: 0.008.

Abbreviations: URL, upper reference limit (0.01 ng/mL); HR, hazard ratio; CI, confidence interval.

Note: Stratification was based on the fold-increase of cardiac troponin T (cTnT) above the upper reference limit. Multifactorial Cox models were adjusted for age, sex, SOFA score, and Charlson comorbidity index.

**Supplementary Table 6. Creatinine-stratified analysis of the association between PLT/LDH ratio and in-hospital mortality**

| **Stratum** | **n** | **HR (95% CI)** | ***p* value** |
| --- | --- | --- | --- |
| T1 (≤80.7 μmol/L) | 753 | 0.673 (0.454-0.999) | 0.049 |
| T2 (80.7-96.6 μmol/L) | 768 | 0.713 (0.468-1.084) | 0.113 |
| T3 (>96.6 μmol/L) | 1,517 | 0.206 (0.124-0.343) | <0.001 |

Interaction *P* value: <0.001.

Abbreviations: HR, hazard ratio; CI, confidence interval.

Note: Of the 3071 patients in the final analysis cohort, 33 had missing serum creatinine data and were excluded from this stratified analysis. The remaining 3038 patients were stratified into tertiles based on serum creatinine. Multifactorial Cox models were adjusted for age, sex, SOFA score, and Charlson comorbidity index

**Supplementary Fig. 1. Patient flow diagram.** A total of 3,390 patients with sepsis-induced myocardial injury (SIMI) were initially screened. After excluding 319 patients with missing platelet count (PLT) or lactate dehydrogenase (LDH) data, 3,071 patients were included in the final analysis cohort, of whom 2,508 survived to hospital discharge and 563 died during hospitalization. Abbreviation: SIMI, sepsis-induced myocardial injury.
